# Treatment of C-Section Scar with Light Touch as a Comparator to Myofascial Release in Patients with Chronic Low Back Pain Case Report

**DOI:** 10.51894/001c.164045

**Published:** 2026-07-31

**Authors:** Nia Kepes, Madison McGath, Joshua Bertman, Angela S. Lee, Austin Waldow, Lauren Gillette, Shivani Anandhasenthil, Kaitlin Howard, Travis Gordon, Jesse Guasco, John M. Popovich Jr.

**Affiliations:** 1 College of Osteopathic Medicine, Michigan State University

## 25

### Introduction

Low back pain (LBP) is prevalent in postpartum women, with cesarean section (CS) considered a significant risk factor. Although clinical guidelines support osteopathic manipulative treatment (OMT) in the management of postpartum patients with LBP, emphasis is largely on treating the lumbar spine and/or pelvis, with lesser attention directed toward the CS scar. Scar-directed interventions are commonly incorporated into post-surgical care, though evidence supporting its direct effects on LBP remains limited.

Light touch, previously considered an inert control, has demonstrated therapeutic effects, and may help clarify the specific clinical contributions of scar-directed OMT. This case report is among the first to evaluate light touch in a unique light and describes the clinical course of the first participant enrolled in an ongoing randomized controlled trial comparing light touch to scar-directed OMT for postpartum LBP following CS delivery, who demonstrated improvements following light touch.

### Case Description

A 35-year-old female, 13-month post-planned CS, presented with persistent LBP that began 1-month post-CS. Her primary concerns were LBP and functional disability. She met the study inclusion/exclusion criteria and was randomized to receive light touch.

Assessments included pain severity (0-10 rating scale), paraspinal stiffness (myotonometry), lumbar range of motion (ROM; inclinometry), and scar mobility (adheremetry). At baseline, LBP was 3/10, paraspinal stiffness averaged 160.3N/m across L4 and L5 spinal levels, lumbar extension ROM was 27º, and scar mobility was restricted (9mm). Following ten minutes of light touch to the CS scar, the immediate effects demonstrated a clinically meaningful improvement (2 points) in LBP to 1/10, as well as documented improvements in paraspinal stiffness (160N/m), lumbar extension ROM (31.5º), and scar mobility (11mm).

### Discussion

This case highlights the therapeutic effects of light touch and supports its use as a comparator to distinguish the effects attributable to touch-based mechanisms from the tissue-specific manipulation components inherent to OMT. As the first participant to receive light touch in our ongoing clinical trial, causality cannot be established. Nevertheless, the observed effects reflect foundational osteopathic principles emphasizing the therapeutic role of touch. Future clinical trial results will provide greater insights into the contributions of touch and the tissue-specific effects of OMT.

